# Chronic Exposure to Uranium from Gestation: Effects on Behavior and Neurogenesis in Adulthood

**DOI:** 10.3390/ijerph14050536

**Published:** 2017-05-17

**Authors:** Céline Dinocourt, Cécile Culeux, Marie Legrand, Christelle Elie, Philippe Lestaevel

**Affiliations:** Laboratoire de RadioToxicologie Expérimentale, Service de Radiobiologie et d’Epidémiologie, Institut de Radioprotection et de Sûreté Nucléaire, B.P.17, 92262 Fontenay aux Roses CEDEX, France; celine.dinocourt@irsn.fr (C.D.); cecile.culeux@gmail.com (C.C.); legmarie@gmail.com (M.L.); christelle.elie@irsn.fr (C.E.)

**Keywords:** hippocampus, brain development, memory, depression, chronic exposure, uranyl, rodent

## Abstract

Uranium exposure leads to cerebral dysfunction involving for instance biochemical, neurochemical and neurobehavioral effects. Most studies have focused on mechanisms in uranium-exposed adult animals. However, recent data on developing animals have shown that the developing brain is also sensitive to uranium. Models of uranium exposure during brain development highlight the need to improve our understanding of the effects of uranium. In a model in which uranium exposure began from the first day of gestation, we studied the neurobehavioral consequences as well as the progression of hippocampal neurogenesis in animals from dams exposed to uranium. Our results show that 2-month-old rats exposed to uranium from gestational day 1 displayed deficits in special memory and a prominent depressive-like phenotype. Cell proliferation was not disturbed in these animals, as shown by 5-bromo-2′deoxyuridine (BrdU)/neuronal specific nuclear protein (NeuN) immunostaining in the dentate gyrus. However, in some animals, the pyramidal cell layer was dispersed in the CA3 region. From our previous results with the same model, the hypothesis of alterations of neurogenesis at prior stages of development is worth considering, but is probably not the only one. Therefore, further investigations are needed to correlate cerebral dysfunction and its underlying mechanistic pathways.

## 1. Introduction

Uranium (U) is an alpha particle-emitting radionuclide of the actinide series and a ubiquitous environmental trace metal found in rocks, soils, water and air [1]. In addition to its geological origin, the increasing role of U in industrial and military processes has resulted in raising occupational exposure to this element. Thus, chronic ingestion of U in drinking water may result in the manifestation of toxicity in many parts of the body [1,2,3]. The central nervous system (CNS) is a target organ for many heavy metals and several reports have described neurological and neurobehavioral effects following internal exposure to U (for a review, see [4]), and more specifically during development. Briefly, U exposure from birth impairs object recognition and memory, causes anxiety and depressive-like behavior in adulthood and disturbs the balance of pro/antioxidant systems [5] and the cholinergic pathway [6]. Studies of animal models show that parental exposure to U during gestation and lactation impairs behaviors such as learning, activity, exploration, and emotionality in offspring [7,8,9]. In our model, in which U exposure began from the first day of gestation, we recently showed depressive-like behavior in neonatal rats, as well as modifications in the signaling pathways of neuronal cell proliferation and neuronal differentiation during prenatal and postnatal development of the hippocampus [10,11]. Using the same model to study specific time points allowed us to follow the neurobehavioral consequences as well as the progression of neurogenesis, from embryogenesis to adulthood in animals from dams exposed to depleted U (DU). Therefore, following our previous studies, we now focus on the effects of chronic exposure to U on neurogenesis in the dentate gyrus of 2-month-old rats. In the first part of the study, the behavior of animals, such as locomotion, memory, anxiety and depression, were evaluated using several tests. Secondly, specific markers of cell proliferation in the hippocampus were assessed by morphological and immunostaining analyses.

## 2. Materials and Methods

### 2.1. Animal Exposure

Twenty-four pregnant female Sprague-Dawley rats (Charles River, L’Arbresle, France) were exposed to U (AREVA, Pierrelatte, France) in drinking water from the first gestational day. The specific activity of U is 14.103 Bq/g and its isotopic composition is 238 U: 99.73%, 235 U: 0.255%. Pregnant rats were exposed to 0, 40, or 120 mg/L, i.e., 0, 2, or 6 mg/kg/day, based on 100 mL of daily water consumption. Thus, rats were exposed to DU throughout life, from embryo to adult stages [10]. For each group (0, 40 and 120 mg/L), 24 pups from eight litters at 2 months of age were taken for analysis. To avoid any effects of hormonal variation in adulthood, we focused our study only on male rats.

All animal procedures were approved by the Institut de Radioprotection et de Sûreté Nucléaire (IRSN) Animal Care Committee and were conducted in accordance with French legislation and European legal requirements (Decree 86/609/EEC) concerning the protection of animals used for experimental purposes. Scientists certified by the French Ministry of Agriculture performed all procedures on the animals.

### 2.2. Behavioral Tasks

The behavioral functions of rats (*n* = 16/group) were evaluated with four commonly used tests: the open-field test, the Y-maze, the elevated plus maze and the Porsolt test, during four consecutive days. The tests were recorded by a video camera and were read by a trainer observer unaware of the exposure conditions.

During the first day of behavioral testing, locomotor and exploratory activities of the rats were assessed in an open field (for methodological details, see [12]). The numbers of horizontal and rearing movements were recorded over a 15-min period. The next day, spatial working memory was assessed in a Y-maze. Each rat was placed at the center of the maze and was allowed to move freely through the maze for a 10-min test session. The sequence and number of arm visits were recorded. Alternation was defined as consecutive entry into each of the three different arms [13]. The third day, anxiety-like behavior was evaluated using the elevated plus maze. Each animal was placed in the center of the maze, facing one of the open arms. The number of entries and the time spent in open and closed arms were recorded for 5 min. The Porsolt test was performed on the fourth day. The rats were placed individually in a glass cylinder, containing water at 23–25 °C, for 10 min. The time of immobility was measured during the last 5 min of the test, as previously described [14].

### 2.3. Morphological Analysis and Neurogenesis

#### 2.3.1. BrdU (5-bromo-2′deoxyuridine) Injection Protocol

In order to evaluate the proliferation of progenitor cells in the hippocampal formation, rats used for behavioral tasks were injected with BrdU (Sigma Aldrich, L’Isle D’Abeau Chesnes, France; 50 mg/kg intraperitoneal (i.p.), dissolved in NaCl) 48 and 24 h before sacrifice.

#### 2.3.2. Tissue Preparation

Rats were anesthetized with an i.p. injection of 60 mg/kg sodium pentobarbital and were perfused transcardially with 4% Paraformaldehyde. After perfusion, brains were post-fixed in the same fixative for 1 h at Room Temperature (RT) and overnight at 4 °C. They were washed in phosphate buffer saline (PBS) 1× solution and then cryoprotected in a solution of 30% sucrose in PBS overnight at 4 °C, quickly frozen on dry ice, and embedded in Tissue-Tek OCT compound, frozen in a liquid nitrogen/isopentane mixture and kept at −80 °C until use. Brains were then sectioned coronally at 40 µm on a cryostat, rinsed in 0.1 M PBS, collected sequentially in tubes containing an ethylene glycol-based cryoprotective solution, and stored at −20 °C until histologic processing.

Every tenth section was stained with cresyl violet in order to determine the general histological characteristics of the hippocampal formation. From each rat brain, adjacent sections were processed for double-labeling immunohistochemistry for the simultaneous detection of BrdU/neuronal specific nuclear protein (NeuN).

#### 2.3.3. Double Immunohistochemical Labeling for BrdU/NeuN

All free-floating sections were first washed in PBS 1×. Sections were incubated in 0.1% trypsin-0.1% CaCl2 diluted in distilled water for 5 min at 37 °C, denatured in 2 M HCl for 30 min and were rinsed twice for 5 min in a borate buffer (55 mL of 0.2 M boric acid/45 mL 0.05 M sodium tetraborate, pH 8.4, Sigma Aldrich, Saint-Quentin Fallavier, France). Sections were rinsed three times in PBS, blocked in PBS containing 0.3% Triton X-100 and 2% milk protein (Regilait, Saint Martin Belle Roche, France) and incubated overnight at 4 °C with a primary mouse monoclonal anti-BrdU antibody (1:50, Dako, Trappes, France) in PBS-Triton-milk. Sections were washed three times in PBS and incubated with cyanine 3-conjugated donkey anti-mouse (1:200, Jackson Immunoresearch, Interchim, Montluçon, France), and diluted in PBS-Triton-milk for 2 h at RT. After washes, sections were incubated with an Alexa-488-conjugated NeuN antibody (1:1000, Millipore, Saint-Quentin Fallavier, France) in PBS-Triton-milk for 1 h at RT, rinsed and mounted with an antifade agent (ProLong Gold, Life Technologies, Villebon-sur-Yvette, France).

#### 2.3.4. Quantification and Image Analysis

In order to perform quantitative analyses in control and exposed animals, four sections from each rat (*n* = 5 control, *n* = 5 DU40 and *n* = 7 DU120 rats) were selected at regular intervals within the rostrocaudal extent of the dorsal hippocampal formation. Analyses were performed by two investigators who were blind to the experimental group treatment. In each section, BrdU-positive cells within the granule cell layer and adjacent subgranular zone were counted through a 40× objective using a fluorescence microscope (Axiophot-Zeiss, Oberkochen, Germany). The density of BrdU-positive cells was calculated by dividing the number of BrdU-positive cells by granule cell layer surface area. For double-staining BrdU/NeuN, percentages of BrdU-positive nuclei co-expressing NeuN were determined by analyzing a minimum of 100 randomly selected BrdU-labeled cells throughout the granule cell layer with a confocal imaging system (LSM 780, Zeiss France, Marly le Roi, France), as previously described by Germain and collaborators [15]. Briefly, BrdU-positive nuclei were analyzed (63× oil objective) in their entire *z*-axis and were rotated in orthogonal planes (*x* and *y*) to verify unequivocally double staining.

### 2.4. Uranium Levels in Tissues

Male rats (*n* = 8/group) were deeply anesthetized with isoflurane 5%/air 95% inhalation and sacrificed by exsanguination. Kidneys and brain were removed in order to measure the concentration of U. All samples were weighed and stored at −20 °C until assay by Inductively Coupled Plasma-Mass Spectrometry (ICP-MS).

Fresh tissue samples weighing 0.48 ± 0.01 g were prepared by adding 8 mL of ultrapure 70% nitric acid and 2 mL of hydrogen peroxide followed by mineralization using a 1000-W microwave (Ethos Touch; Milestone Microwave Laboratory Systems; Sorisole, Italy) with a 20-min ramp at 180 °C, followed by 10 min at 180 °C. U content from mineralized samples was determined using an inductively coupled plasma mass spectrometer (ICP-MS-VGPQ, EXCELL, ThermoElectron, Saint-Herblain, France) with bismuth (1 µg/L) as an internal standard. For U, the ICP-MS limit quantification is 10^−3^ µg/L.

### 2.5. Statistical Analysis

Data from behavioral tests are expressed as mean ± standard deviation (SD) and were analyzed by two-way analysis of variance (ANOVA) with the main factors of group and dose. Post-hoc comparisons were made with the Student-Newman-Keuls test.

Body weights of male rats are presented as mean ± SD in Table 1. One-way ANOVA followed by Dunn’s method or the Holm-Sidak method was used to compare the body weights of the control and exposed groups. U concentrations in samples were expressed as nanogram per gram of fresh tissue and presented as mean ± SD. They were compared between control and DU-exposed groups with one-way ANOVA followed by Tukey’s test (Table 1).

Immunochemistry data were presented as means ± SD. One-way ANOVA followed by Dunn’s method or the Holm-Sidak method was used to compare the control and exposed groups.

Differences were considered statistically significant for *p* < 0.05 or *p* < 0.01. All statistical analyses were performed using Sigmaplot software (Systat software Inc., San Jose, CA, USA) by using standard deviation (SD).

## 3. Results and Discussion

### 3.1. General Health Parameters and U Concentrations in Tissues

U concentrations in the kidneys and brain of 2-month-old male rats were significantly higher in rats exposed to 40 and 120 mg/L U than in control animals, without significant modification of drinking water quantities and body weight (Table 1). The concentrations of U in rats exposed to 120 mg/L were significantly higher (around three times as high) in comparison with the 40 mg/L group (Table 1). These results are in line with the data of Legrand and collaborators [10], and together demonstrate that the dose-dependent accumulation of U occurred in the bodies of pups from postnatal day 0 until adulthood throughout exposure.

### 3.2. Behavior

Developing animals, i.e., during the pre- and postnatal periods, are the most sensitive to the effects of U [16,17]. The neurological effects of U during these periods might induce severe behavioral impairments in adulthood. Behavioral tests were thus performed on 2-month-old male rats from dams exposed to 40 or 120 mg/L U from gestational day 1.

**Figure 1:**
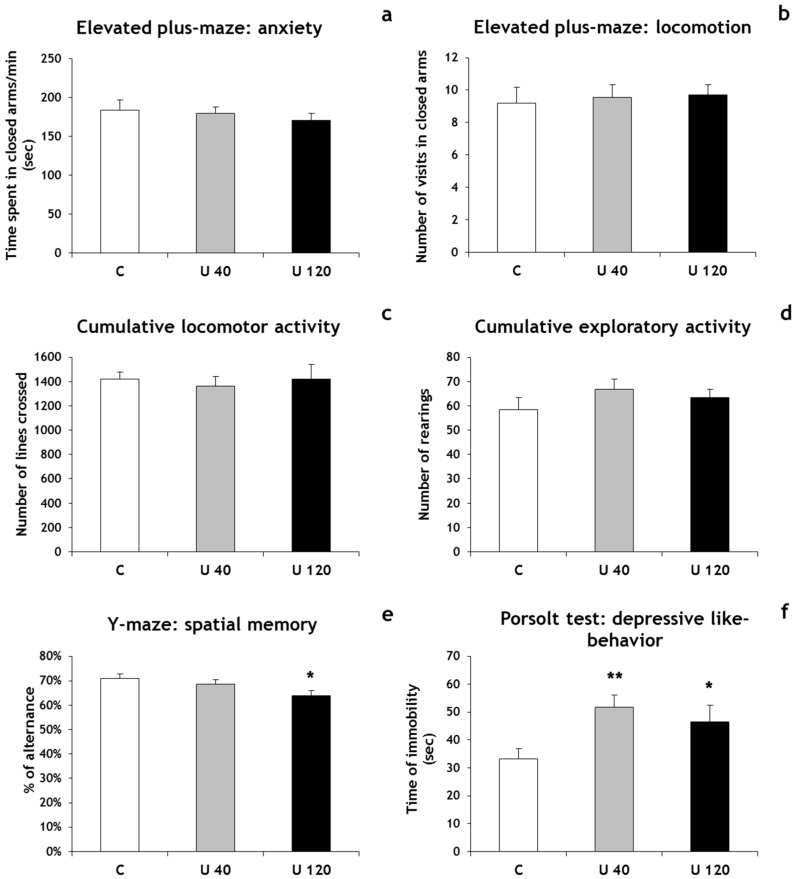
Behavioral parameters measured in control rats and rats exposed to U from gestational day 1 and until 2 months of age. (**a**) Time spent in the closed arms of the elevated plus maze expressed in seconds that reflects the anxiety level; (**b**) Number of visits in closed arms of the elevated plus maze that reflects locomotor activity; (**c**) Number of lines crossed, reflecting locomotor activity; (**d**) Number of rearings that reflects exploratory activity; (**e**) Percentage of alternation of the Y-maze that reflects the spatial working memory; (**f**) Time of immobility in seconds of the Porsolt test that reflects the depression level. Results are expressed as mean ± SD; *n* = 16/group; ** *p* < 0.01 or * *p* < 0.05 indicate significant difference from the control value. U 40: group 40 mg/L; U 120: group 120 mg/L.

To investigate anxiety-like behavior, animals were tested in the elevated plus maze. The percentage of time spent in the closed arms of the elevated plus maze did not significantly differ between exposed animals and controls (Figure 1a). Additionally, no differences were found in the distance travelled in the elevated plus maze, suggesting that locomotor behavior was not affected by U exposure in our experimental conditions (Figure 1b). This point was confirmed by the open-field test, which did not reveal significant differences in locomotor and exploratory activities between U-exposed rats and controls in our conditions (Figure 1c,d). The Y-maze test was used to evaluate spatial working memory and showed that the percentage of alternation decreased significantly between rats exposed to 120 mg/L DU and control groups (−10%, *p* < 0.05), but no significant difference was observed between the 40 mg/L and control groups (Figure 1e). Finally, the Porsolt swim test showed a significant increase in immobility time in rats exposed to 120 mg/L U (+40%, *p* < 0.05) as well as 40 mg/L U (+56%, *p* < 0.01), compared with non-exposed rats (Figure 1f).

Thus, U exposure from gestational day 1 induced memory deficit and a prominent depressive-like phenotype in adulthood. These behavioral results are in accordance with our previous article demonstrating no significant effect on locomotor activity, a significant decrease of working memory and an induction of depressive like-behavior in postnatal day 21 rat pups from U-exposed dams [11]. Other studies have also demonstrated that exposure to U during development affects behavioral responses. Rodents exposed to U or 4% enriched U during gestation and lactation showed a significant decrease in short-term memory and/or a change of locomotor activity at the adult age [7,8]. When the exposure to U starts at birth, spatial working memory is impaired and depressive-like behavior is observed [5]. All these results show that exposure to U early in life could have potential delayed effects in adulthood. These effects of U on memory could result from the fact that several brain structures, such as the hippocampus and the entorhinal cortex, are the substrates for memory. It has been suggested that impairment in hippocampal function may be also involved in the etiology of depression [18]. Additional tests, such as the Morris water maze, the sucrose preference test and the tail suspension test, are necessary to further investigate the potential effects of developmental U exposure on cognitive and mood disorders.

### 3.3. Hippocampal Morphology and Neurogenesis

#### 3.3.1. Hippocampal Adult Neurogenesis Is Normal in U-Exposed Rats

**Figure 2:**
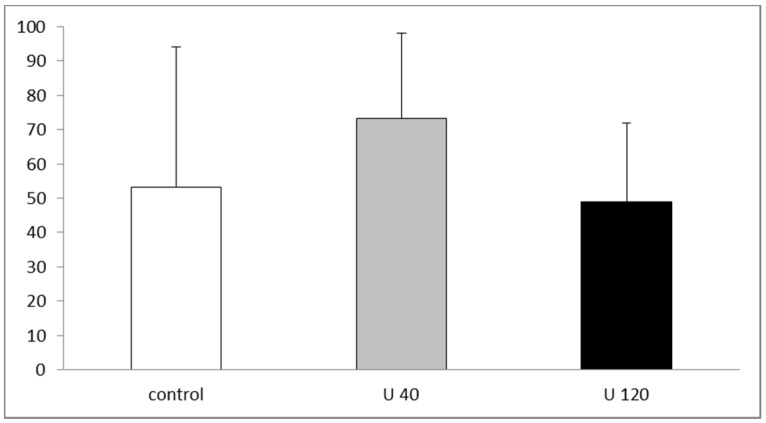
Cell proliferation in 2-month-old control rats and rats exposed to U from gestational day 1. Ratio of BrdU-positive cell number to total area (BrdU positive cells/mm^2^) in the granular cell layer of the dentate gyrus. U40: group 40 mg/L; U120: group 120 mg/L.

Proliferation of hippocampal newborn granule cells was quantified in 2-month-old rats from the U-exposed and control dams. Our data revealed that U-exposed rats exhibited similar numbers and spatial distribution of BrdU-positive proliferative cells as compared with controls (Figure 2). In addition, we quantified the proportion of BrdU/NeuN co-labeling cells. We used confocal microscopy to count the percentage of double- or single-labeled BrdU-positive cells in the dentate gyrus (DG) and found no statistical difference between exposed and non-exposed groups (control: 95.1%; U 40: 94.9%; U 120: 95.6%; Figure S1). Based on these results, cell proliferation and neuronal differentiation seemed to be preserved in exposed animals as compared with controls.

The study of specific time points in our model of U exposure from gestation day 1 allowed us to follow the progression of neurogenesis and more specifically the proliferative state by BrdU incorporation in the DG, from embryogenesis to adulthood in animals from the U-exposed dams ([10,11] and present work). In summary, no significant change was observed after U exposure during the development of the DG at gestational day 13, postnatal day 0 and day 5 [10], as well as at 2 months (present study). However, U exposure increased cell proliferation by increasing the production of dividing neural progenitors in the dentate neuroepithelium of fetuses [10]. Correlated with this, exposure to U seems to promote neural cell proliferation during prenatal development [11]. In addition, we showed that the number of BrdU-positive cells in the granule cell layer of 21 postnatal rats from dams exposed to 120 mg/L U was significantly decreased [10]. Thus, even though U targets cell proliferation in the DG, its effects were not visible throughout exposure.

**Figure 3:**
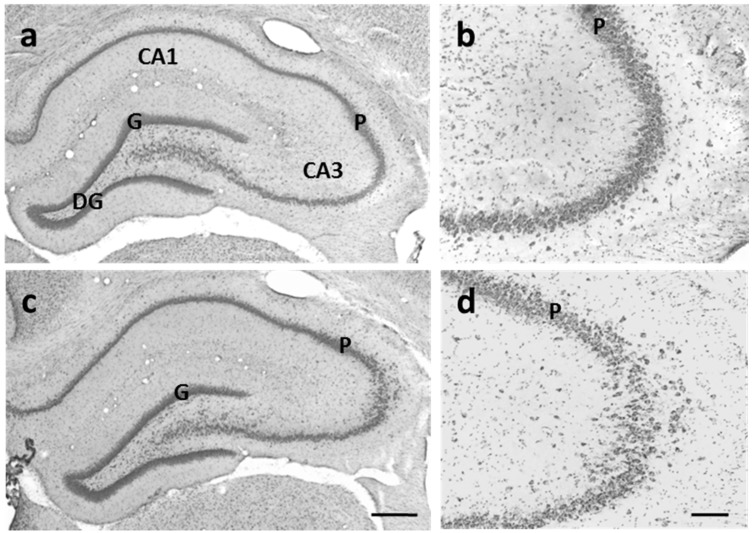
Cresyl violet-stained sections of the hippocampal formation of 2-month-old control rats (**a**,**b**) and 2-month-old rats exposed to U (**c**,**d**) since gestational day 1. (**a**) In a control animal, the cell bodies of principal cells are concentrated and form a continuous band in the pyramidal cell layer (P) in the CA3 region and in the granule cell layer (G) of the dentate gyrus; (**b**) High-magnification photomicrographs of the CA3 region; (**c**) In an exposed rat, the cell bodies of principal cells are concentrated and form a continuous band in the granule cell layer (G) of the dentate gyrus, like the control animal, but are dispersed in the CA3 region; (**d**) High-magnification photomicrographs of the CA3 region. Scale bars: 500 µm in (**a**,**c**) and 100 µm in (**b**,**c**).

DU exposure did not induce major alterations in the organization of the DG, as seen in the organization of the granule cell layer in 2-month-old rats (Figure 3). Also, no major organogenesis or developmental disturbance was observed in the development of the DG [10]. Nevertheless, the fact that there was no change in the laminar organization of cell bodies or in the spatial distribution of BrdU-positive cell in the DG during its development did not mean that there were no changes in the process of proliferation or in neuronal differentiation that might disrupt the fine organization of the neuronal network and alter synaptic integrity in the DG. To support this, we observed changes in the expression of doublecortin, a microtubule-associated protein, in immature neurons in the granule cell layer of postnatal day 21 rats of U-exposed dams [11] as well as in adult rats (personal communication).

#### 3.3.2. CA3 Pyramidal Cells are Dispersed in U-Exposed Rats

As described above, laminar organization of the DG was not modified in exposed animals. Likewise, the CA1 and CA2 regions showed normal lamination. However, cresyl violet staining revealed that CA3 pyramidal cells were abnormally dispersed or disrupted in the CA3 region of exposed rats (*n* = 2/5 U40 and *n* = 5/7 U120) compared with the single diffuse layer of non-exposed rats (Figure 3). Such abnormalities were observed at all levels in the hippocampus along the rostrocaudal axis. Brain malformations and developmental variations in fetuses, such as exencephaly at high U doses, have been previously described [16]. In our experimental conditions, these types of malformations were never observed. Nevertheless, this abnormal cell distribution in the CA3 region showed that the process of cell migration could be impaired by U exposure during brain development. In support of this hypothesis, abnormal lamination of the CA3 region was locally observed in animals exposed to U from birth to 2 months [19].

Taken together, these initial results confirm the need for the further investigation of other markers of cell proliferation and neuronal differentiation and of other steps of cell migration and synaptogenesis, if we are to conclude unequivocally that U exposure impairs the fine organization of the neuronal network by altering neurogenesis during brain development and might alter brain functions in adulthood.

## 4. Conclusions

In conclusion, our results show that 2-month-old rats exposed to U from gestational day 1 displayed deficits in special memory and a prominent depressive-like phenotype. Based on the present data from BrdU/NeuN immunostaining analyses, adult neurogenesis in the DG does not seem to be associated with these impairments.

From our present and previous data, several hypotheses might be formulated to correlate these neurobehavioral impairments with potential mechanistic pathways: (a) the alterations observed in the process of neurogenesis during development [10,11]; (b) a disruption of the fine organization of the neuronal network and modification of synaptic integrity in the DG during development as well as in adulthood; (c) neurogenesis alone is not sufficient to induce these behavioral impairments. To support the latter hypothesis, Lestaevel and collaborators have previously demonstrated that U exposure from birth alters oxidative stress responses, metabolism of acetylcholine pathways as well as the metabolite profile in the cerebrospinal fluid in animals with impairment of neurobehavioral function [5,6,20].

Long-term low-level U exposure is a hazard to human health, particularly for pregnant women and children. Thus, further investigations are needed to correlate brain dysfunction and its underlying mechanistic pathways during the sensitive early life stages.

## Figures and Tables

**Table 1:** Health indicators and U concentration measured in control rats and rats exposed to U from gestational day 1 and until 2 months of age.

		Control	U 40	U 120
**Health indicators**	Weight (g)	336.6 ± 31.7	343.0 ± 30.8	352.4 ± 40.1
(*n* = 16/group)	Food (g/day/rat)	27.8 ± 0.7	28.1 ± 0.4	28.0 ± 0.5
	Water (mL/day/rat)	27.7 ± 0.7	28.0 ± 1.2	28.7 ± 1.0
**U concentration**	Kidneys (ng/g)	6.5 ± 0.7	193.3 ± 21.8 *	584.5 ± 73.3 *
(*n* = 8/group)	Brain (ng/g)	0.10 ± 0.02	0.20 ± 0.02 *	0.67 ± 0.13 *

Results are expressed as mean ± SD. * *p* < 0.05 is significantly different from the control value. U40: group treated with 40 mg/L U daily; U120: group treated with 120 mg/L U daily.

## Supplementary Materials

The following are available online at www.mdpi.com/1660-4601/14/5/536/s1, Figure S1: Cell proliferation in 2-month-old control rats and rats exposed to U from gestational day 1.
