# Supplementary material for: Chronic Exposure to Uranium from Gestation: Effects on Behavior and Neurogenesis in Adulthood
Source: Int J Environ Res Public Health. 2017 May 17;14(5):536. doi: 10.3390/ijerph14050536 (PMC5451987; doi:10.3390/ijerph14050536)

Figure S1. Cell proliferation in 2-month-old control rats and rats exposed to U from gestational day 1. Confocal images of BrdU/NeuN immunostaining in the dentate gyrus of control (**a**) and U (120 mg/L, **b**) exposed dams. In green: NeuN-positive cells; in red BrdU-positive cells (arrow) and in yellow BrdU/NeuN-positive cells (head of arrow). Scale bar 100 μm.

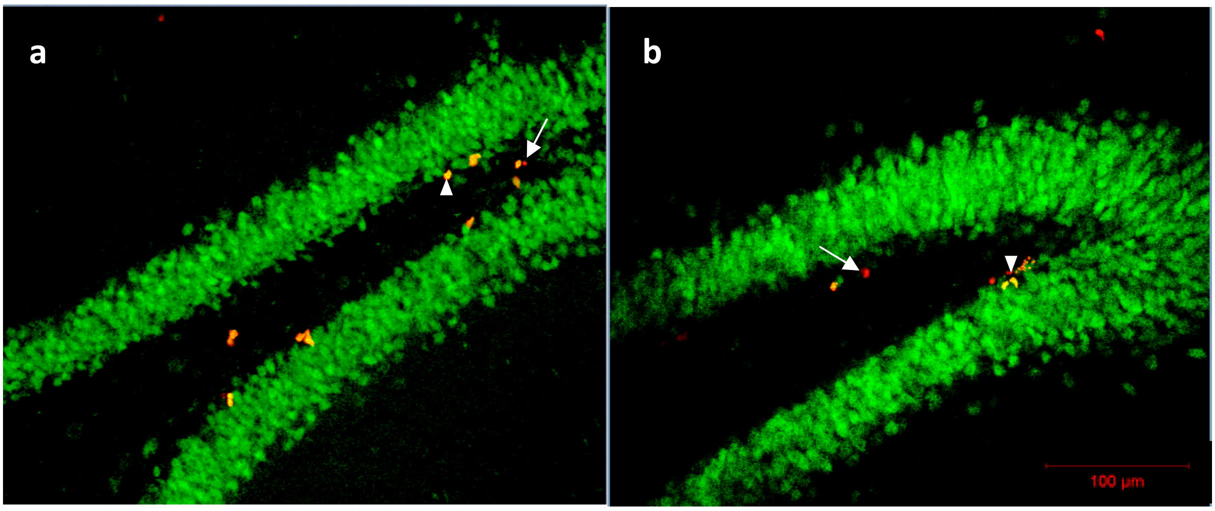

Supplement: Supplementary file 1 [file ijerph-14-00536-s001.pdf]
